# Supplementary material for: Bacterial chromosomal mobility via lateral transduction exceeds that of classical mobile genetic elements
Source: Nat Commun. 2021 Nov 8;12:6509. doi: 10.1038/s41467-021-26004-5 (PMC8575950; doi:10.1038/s41467-021-26004-5)
Supplement: Supplementary file 2 — Reporting Summary [file 41467_2021_26004_MOESM2_ESM.pdf]

nature research

Corresponding author(s): José R. Penadés

Last updated by author(s): Aug 25, 2021

Reporting Summary

Nature Research wishes to improve the reproducibility of the work that we publish. This form provides structure for consistency and transparency in reporting. For further information on Nature Research policies, see Authors & Referees and the Editorial Policy Checklist .

Statistics

For all statistical analyses, confirm that the following items are present in the figure legend, table legend, main text, or Methods section.

n/a	Confirmed	
x	☐	The exact sample size (*n*) for each experimental group/condition, given as a discrete number and unit of measurement
x	☐	A statement on whether measurements were taken from distinct samples or whether the same sample was measured repeatedly
x	☐	The statistical test(s) used AND whether they are one- or two-sided<br>*Only common tests should be described solely by name; describe more complex techniques in the Methods section.*
x	☐	A description of all covariates tested
x	☐	A description of any assumptions or corrections, such as tests of normality and adjustment for multiple comparisons
x	☐	A full description of the statistical parameters including central tendency (e.g. means) or other basic estimates (e.g. regression coefficient) AND variation (e.g. standard deviation) or associated estimates of uncertainty (e.g. confidence intervals)
x	☐	For null hypothesis testing, the test statistic (e.g. *F*, *t*, *r*) with confidence intervals, effect sizes, degrees of freedom and *P* value noted<br>*Give P values as exact values whenever suitable.*
x	☐	For Bayesian analysis, information on the choice of priors and Markov chain Monte Carlo settings
x	☐	For hierarchical and complex designs, identification of the appropriate level for tests and full reporting of outcomes
x	☐	Estimates of effect sizes (e.g. Cohen's *d*, Pearson's *r*), indicating how they were calculated

Our web collection on statistics for biologists contains articles on many of the points above.

Software and code

Policy information about availability of computer code

Data collection	DNA reference sequences were obtained from NCBI, Genbank (https://www.ncbi.nlm.nih.gov/nucleotide/)
Data analysis	SnapGene Viewer version 5.3 (open source software: https://www.snapgene.com/snapgene-viewer/) was used to import and visualise DNA sequences from Genbank for different genetic elements where accession numbers were available, to enable determination of the total number of ORFs and their predicted functions for calculation of estimated 'cargo capacity' rates. The accession numbers are provided in the manuscript in Tables 1 and 3.<br><br>SnapGene Viewer version 5.3 was used to import and visualise complete chromosomal DNA sequences for S. aureus NCTC8325-4 (accession: NC_007795.1) and S. Typhimurium LT2 (accession: AE006468.2) to enable:<br>i) mapping of the coordinates of successive lateral transducing particle headfuls from each attB site;<br>ii) estimation of the number of ORFs contained within each lateral transducing particle headful for calculation of estimated 'cargo capacity';<br>iii) location mapping of loci/genes of interest relative to attB sites on the S. aureus and S. Typhimurium chromosomes.

For manuscripts utilizing custom algorithms or software that are central to the research but not yet described in published literature, software must be made available to editors/reviewers. We strongly encourage code deposition in a community repository (e.g. GitHub). See the Nature Research guidelines for submitting code & software for further information.

Data

Policy information about availability of data

All manuscripts must include a data availability statement. This statement should provide the following information, where applicable:

- Accession codes, unique identifiers, or web links for publicly available datasets
- A list of figures that have associated raw data
- A description of any restrictions on data availability

Source data is available in the accompanying excel file. For all data derived from published literature, the source is indicated as a citation in the manuscript. Accession numbers relating to each element analysed are included in the manuscript (Tables 1 and 3) where they were available.

Field-specific reporting

Please select the one below that is the best fit for your research. If you are not sure, read the appropriate sections before making your selection.

- [x] Life sciences
- [ ] Behavioural & social sciences
- [ ] Ecological, evolutionary & environmental sciences

For a reference copy of the document with all sections, see nature.com/documents/nr-reporting-summary-flat.pdf

Life sciences study design

All studies must disclose on these points even when the disclosure is negative.

Sample size	No calculations for determining sample size were required for this study as statistical comparisons were not made between the different elements analysed.
Data exclusions	'Cargo capacity' and 'relative frequency of genetic mobility' rates were not calculated for elements where there was insufficient sequence data or a lack of information in the source literature to enable determination of the total number and/or putative function of ORFs carried by the element. These instances are denoted as 'ND' (not determined) in Tables 1 and 3.
Replication	Experiments were performed at least three times as independent biological replicates. All attempts at replication were successful. Information regarding the number of independent replicates performed for each genetic element (i.e. phage, plasmid, etc) are included in the methods section of the manuscript, as well as in the footnotes of Table 2.
Randomization	Randomization was not relevant to this study as the genetic elements (i.e. phages, plasmids, etc) that were compared had clearly defined differences in their mobility mechanisms. In addition, the data used in the analysis was sourced from a variety of published literature sources, as well as new experimentally-derived data, precluding randomization in the analysis.
Blinding	Blinding was not relevant to this study as different mechanisms of gene transfer (with clearly defined differences) were compared.

Reporting for specific materials, systems and methods

We require information from authors about some types of materials, experimental systems and methods used in many studies. Here, indicate whether each material, system or method listed is relevant to your study. If you are not sure if a list item applies to your research, read the appropriate section before selecting a response.

Materials & experimental systems

n/a	Involved in the study
☒	☐ Antibodies
☒	☐ Eukaryotic cell lines
☒	☐ Palaeontology
☒	☐ Animals and other organisms
☒	☐ Human research participants
☒	☐ Clinical data

Methods

n/a	Involved in the study
☒	☐ ChIP-seq
☒	☐ Flow cytometry
☒	☐ MRI-based neuroimaging
